# Our Sons and Daughters

**Published:** 1869-12

**Authors:** 


### HALL'S JOURNAL OF HEALTH.

OUR LEGITIMATE SCOPE IS ALMOST BOUNDLESS: FOR WHATEVER BEGETS PLEASURABLE AND HARMLESS FEELINGS, PROMOTES HEALTH; AND WHATEVER INDUCES DISAGREEABLE SENSATIONS, ENGENDERS DISEASE.

*We aim to show how Disease may be avoided, and that it is best, when sickness comes, to take no Medicine without consulting an educated Physician.*

VOL. XVI.] DECEMBER, 1869. [No. 12.

### OUR SONS AND DAUGHTERS.

"LITTLE LIBBIE has been four years in heaven to-day. Our nurse sent her there when she was but a year and a quarter old, by feeding her with a parcel of green grapes, which brought on a fatal disease of the bowels." Such was the narration of a friend of ours a few moments ago, this beautiful first of November morning.

Multitudes of other children are slaughtered every year by the ignorant, blarneying, and unprincipled generation which officiates as nurse for our children. If a man wants to raise a fine pig, he sees to it himself, that it it is properly fed every day, or appoints one who knows something of the nature and needs of pigs. If a stock raiser wants to rear a stallion, or a "pair," for his own use, or "for a market," his groom is selected in reference to his experience and knowledge. But when the infant of our heart is to be watched over and cared for, which is to be a blessing or a burden for a life-time to itself, to ourselves, and to friends, we select a trifling girl, hardly yet in her teens, as being "good enough for a nurse," and as costing less wages, by a dollar or two a month, eating more than that difference, and wasting five times as much. Or if we do not manage it in that way, we go to some ship just arrived, with its cargo of vermin-swarming, semi-idiotic, poverty-stricken unfortunates, in the vain hope of rearing them to our notion; and our blessed children are given them as materials to try their hand upon, just as if a diamond of the first water was given to a prentice boy, just brought into the shop, to learn him the mystery and art of fitting it for the casket of a king. It is enough to make one boil with rage, in passing near or through our parks and better thoroughfares, to see how the smallest children are set down on the grass, or stone steps, or slung across gutters by one arm, or compelled to travel homeward, after being wearied with being abroad for several hours, their piteous cries of "tarri me, nursy" being as unheeded as the wail of a human in the jaws of a shark.

We need not wonder that, with such attention, multitudes of children do worse than die—grow up in deformity of person, or blighted in heart, or ruined in constitution, the penalty being a weary life-time of pain.

What is the remedy? Humanity, reason, religion, in one clear, loud voice, in accents fearfully urgent, cry out: "It is within ourselves." We must begin by divesting ourselves of that inexcusable ignorance, that contemptible pride, that degrading deference to "what will Mrs. Grundy say," to the opinion of our neighbors, which binds in manacles of brass the "sovereigns" of this great nation. Talk about every citizen of the United States being a "sovereign." Half of us are slaves. Not slaves to one another, but the slaves of our cooks and house-maids and seamstresses and footmen and nurses—slaves too, to the opinion of our neighbors, and there is no tyranny on earth like it, more remorseless, more exacting. Pretty Mrs. SMITH, a brewer's daughter, will not measure out the tea and coffee, because "it looks mean," and the cook will talk about it the next place she goes to. And for the same reason, she allows all the "scraps," to be thrown into the ash-barrel—no half loaf, or remnant of pudding or pie must appear on her table. She wants to be thought generous and liberal, and orders these things to be given to vagrant children and abandoned girls and drunken loafers, who besiege our gates like hordes of vultures.

Most generally, it is only "stuck up" people who reason like the brewer's daughter, while those who are conscious of their own high position, never busy themselves in calculating what other people will say, or in arranging their conduct in such a manner as to be best adapted to make a favorable impression on others. On the contrary, they are satisfied with the purity and rationality of their motives, and hence, are contented to let their actions take care of themselves. It is only the "fency" politician, the tainted character, or the "dubious," who are always defining their positions, such as they are! Again, we inquire, "what is the remedy?" Let the mothers of this great land remember, that it is impossible for every daughter to marry a man who is able to support her in idleness. Let them remember, that not one daughter in a hundred thousand, will marry thus. Let mothers remember, that of every hundred daughters, not over one will escape for the first thirty years of life, the necessity of contriving, of resorting to expedients, of pinching economies, in order to keep up appearances, or barely make both ends meet; or of doing these things at the expense of others, who, by thrift, economy and industry, had a plenty for themselves and a heart to help a friend in need. For the truly generous are they, whose lot it was to help themselves.

Within a month, we chanced to spend a night with a family a hundred miles away, up the Hudson. Before we entered the house, we were satisfied that there was comfort, abundance, and thrift there. We found that father and mother at threescore and ten, were hale and hearty, and were born there. Sons and daughters were grown up. Such groaning tables, such beds and bedding, such tidiness, such spotless cleanliness, in hall and parlor and dining room, everywhere. "No servant curses this household," thought we. And it was so. Sons and daughters, old people and young, all worked, and delighted in it, for the positive comfort it gave them, for the independence and for the exemption it afforded from that ceaseless watching, that endless repetition of directions, and that never absent uncertainty, as to whether orders have been attended to, which all together join in eating out the sweets of domestic life in this tyrant-ridden "free country."

Let mothers remember, that there is no guarantee against poverty, want, and abject destitution in this nation, for a single generation. That not one in a hundred of all the girls in this land will escape the necessity of sewing, patching, cooking and washing for themselves. Does not then your own common sense teach you, that it is better they should learn these things under your own affectionate teaching, under your own leisure roof, with all the appliances of a well-ordered household, where it can be done without waste, without anxiety, without a heart almost broken by disappointment and a hard lot in life?

A million dollars reward might be safely offered to the man who could produce a single family in all this broad land, which was the worse for having had to do their own work in early life, while tens of thousands of families are there, which are the worse, both in position and purse, from having been brought up to feel that it was a disgrace to scrub the floor, to patch a pair of pantaloons, or wash the clothes. Many years ago, there lived a family of growing up children on one of the farms of Northern New York. There were four daughters and two sons. For a long time, they had no servant, but as the family grew up, one servant was necessary to do the washing. On such occasions, the mother and daughters were up betimes, and had the water heated, and tubs prepared and clothing ready, and while one daughter dusted up the house, and another prepared the breakfast, the others, with their mother, stood in their lot beside the servant, and while they washed, they, at the same time, saw to it, that the "hired help" earned her wages. As to the sons, they worked in summer and went to school in winter, and out of school hours, did the "chores," fed the pigs, cut the wood, gathered the kindlings and brought in all the cobs, "that "sisters" might kindle the kitchen fire easily, and that "mother" might not have to go out of doors at daylight in the bitter cold. The result was, the sons grew up to feel that they must work for a living, and soon, there came in a nobler sentiment, that manly independence, which grows out of a conviction that a living can be made.

As to the daughters, it is true, they baked bread, washed the floors, darned stockings, sewed on buttons, and did whatever was to be done; and being rid of the intolerable arrogance of slattern servants, there was constant sunshine in their house, in their hearts, and in their faces, while upon the whole mein and character, there was a self-consciousness of worth, and of independence, which never fails to command the respect of all who come in contact with the possessor of these.

These girls were not caught up in a hurry. The scum and flitting birds were not at all attracted to girls at the wash-tub, but in due time, they all married men. One is the mother of children, who are foremost in wealth and influence in the large city where they live. A second did just as well in another large city, and a third likewise. The fourth and eldest, is the mother of four children, the wealth of each of whom, in New York city, is counted by millions, literally. As to the two sons, both are rich, within ten miles of the City Hall, with large families, and not one black sheep among them. The son of one is nearing a professional eminence and success, equal to the highest; and the son of the other, is worth a million of money in Wall street, and his name good for millions more. Who then shall say, that any of these were the worse for the wash tub, the scrubbing brush, the kitchen, or the hoe handle? And yet, a large proportion of parents in our country, have an abiding impression, that there is degradation in these things, and the mother takes the broom out of her daughter's hands, and the father grubs in his blouse, while the son sports a moustach or imperial and promenades in gloves and broad-cloth.

Girls have risen before now, from the wash-tub to the throne, and elevated both. The perfect master of a calling elevates it. No essentially useful occupation can degrade the man who thoroughly understands his business; it only helps him to rise above it, until he reaches a position which is adequate to his capabilities.

"After the death of his wife, Sir Charles Napier removed to Caen, in Normandy, and did his best to perform the part of mother to his girls. His aim was to make them religious, as the foundation of all excellence; to teach them accounts, that they might learn the value of money; work, that they might not waste their time if they were rich, nor be helpless if they were poor; cooking, that they might guard against the waste of servants, and be able to do for themselves in the event of a revolution."

There is not a girl on earth, whether the daughter of prince or pauper, who, if made a perfect mistress of all household duties, and were thrown into a community wholly unknown, would not rise from one station to another, and eventually become the mistress of her own mansion, while multitudes of young women, placed in positions of ease, elegance, and affluence, but being unfitted to fill them, will as certainly descend from one round of the ladder to another, until at the close of life, they are found where the really competent started from.

Mothers of America, if you wish to rid your own and your childrens' households of the destroying locusts which infest your houses and eat up your substance, take a pride in educating your daughters to be perfect mistresses of every home duty; then, even if you leave them without a dollar, be assured, they will never lack a warm garment, a bounteous meal, or a cosy roof, nor fail of the respect of any who know them.

